# Cranberry Extract for Symptoms of Acute, Uncomplicated Urinary Tract Infection: A Systematic Review

**DOI:** 10.3390/antibiotics10010012

**Published:** 2020-12-25

**Authors:** Oghenekome A. Gbinigie, Elizabeth A. Spencer, Carl J. Heneghan, Joseph J. Lee, Christopher C. Butler

**Affiliations:** Nuffield Department of Primary Care Health Sciences, University of Oxford, Radcliffe Primary Care Building, Radcliffe Observatory Quarter, Woodstock Road, Oxford OX2 6GG, UK; elizabeth.spencer@phc.ox.ac.uk (E.A.S.); carl.heneghan@phc.ox.ac.uk (C.J.H.); joseph.lee@phc.ox.ac.uk (J.J.L.); christopher.butler@phc.ox.ac.uk (C.C.B.)

**Keywords:** cranberry, vaccinium macrocarpon, herbal, urinary tract infection, systematic review, antibiotic resistance

## Abstract

Background: Effective alternatives to antibiotics for alleviating symptoms of acute infections may be appealing to patients and enhance antimicrobial stewardship. Cranberry-based products are already in wide use for symptoms of acute urinary tract infection (UTI). The aim of this review was to identify and critically appraise the supporting evidence. Methods: The protocol was registered on PROSPERO. Searches were conducted of Medline, Embase, Amed, Cinahl, The Cochrane library, Clinicaltrials.gov and WHO International Clinical Trials Registry Platform. We included randomised clinical trials (RCTs) and non-randomised studies evaluating the effect of cranberry extract in the management of acute, uncomplicated UTI on symptoms, antibiotic use, microbiological assessment, biochemical assessment and adverse events. Study risk of bias assessments were made using Cochrane criteria. Results: We included three RCTs (*n* = 688) judged to be at moderate risk of bias. One RCT (*n* = 309) found that advice to consume cranberry juice had no statistically significant effect on UTI frequency symptoms (mean difference (MD) −0.01 (95% CI: −0.37 to 0.34), *p* = 0.94)), on UTI symptoms of feeling unwell (MD 0.02 (95% CI: −0.36 to 0.39), *p* = 0.93)) or on antibiotic use (odds ratio 1.27 (95% CI: 0.47 to 3.43), *p* = 0.64), when compared with promoting drinking water. One RCT (*n* = 319) found no symptomatic benefit from combining cranberry juice with immediate antibiotics for an acute UTI, compared with placebo juice combined with immediate antibiotics. In one RCT (*n* = 60), consumption of cranberry extract capsules was associated with a within-group improvement in urinary symptoms and Escherichia coli load at day 10 compared with baseline (*p* < 0.01), which was not found in untreated controls (*p* = 0.72). Two RCTs were under-powered to detect differences between groups for outcomes of interest. There were no serious adverse effects associated with cranberry consumption. Conclusion: The current evidence base for or against the use of cranberry extract in the management of acute, uncomplicated UTIs is inadequate; rigorous trials are needed.

## 1. Introduction

Women often experience symptoms attributed to urinary tract infection (UTI) [1] and a high proportion receive antibiotic treatment [2]. Increasing antibiotic resistance has sparked interest in non-antibiotic treatments for common bacterial infections, such as UTIs [3,4,5,6]. 

Cranberry fruit (Vaccinium macrocarpon) grows on evergreen shrubs that are native to North America [7]. Cranberry fruit is classed as a functional food due to the naturally high content of compounds, such as polyphenols, which are believed to have antioxidant and therefore health-promoting properties [8]. The reported health benefits of cranberry consumption range from cardioprotective effects due to improved cholesterol profiles [9] to aiding digestive health [10]. Cranberry exists in various forms, including the raw fruit (fresh and dried), cranberry juice and cranberry extract in capsule/tablet formulations [11]. 

Cranberry extract could be a potential alternative to antibiotics to treat acute uncomplicated UTIs. Proanthocyanidin (PAC) with A-type linkages, or their metabolites, are believed to be the active ingredient in cranberry, preventing *Escherichia coli* (*E. coli*) from binding to the bladder uroepithelium [12] and thereby reducing the ability of E. coli to cause and sustain a UTI. Systematic reviews assessing the use of cranberry in the management of recurrent UTIs provide mixed evidence for benefit [13,14]. A 2012 Cochrane review of 24 trials (*n* = 4473) of men, women and children found that cranberry did not significantly reduce recurrent UTI compared with placebo, advice to increase water intake or no treatment. A subgroup analysis of women with recurrent UTI found that cranberry consumption resulted in a non-significant reduction in recurrent UTIs [15].

Whilst many studies have evaluated the effectiveness of cranberry extract in reducing recurrent UTI, few have assessed effects on symptoms of acute UTIs [16]. The aim of this systematic review was therefore to synthesise the evidence for the use of cranberry products in the management of acute, uncomplicated UTIs.

## 2. Methods

The protocol was registered on the PROSPERO database on the 13th March 2020 (https://www.crd.york.ac.uk/prospero/display_record.php?RecordID=166785). 

### 2.1. Search Strategy

We conducted searches in Medline, Embase, The Cochrane Library, Amed, Web of Science and Cinahl from inception to 3rd February 2020. We also searched ClinicalTrials.gov, the WHO International Clinical Trials Registry Platform (ICTRP) and Google Scholar for relevant studies. Search terms included, but were not limited to, cranberry, vaccinium and urinary tract infection (see Table S1 for the comprehensive search strategy). There were no language or time restrictions. We consulted experts in the area and manufacturers of cranberry products (Ocean Spray and Trophikos, LLC) to identify relevant studies, and the bibliography of selected articles was hand-searched to find further eligible studies. Manufacturers of cranberry products had no other involvement in this systematic review. Each citation was independently assessed for eligibility by two reviewers (OAG and either JJL or EAS), with disagreements resolved by discussion. 

### 2.2. Eligibility Criteria and Study Selection

We included RCTs (blinded and open-label) comparing the effectiveness of cranberry extract with any other treatment for acute uncomplicated UTIs in patients aged 18 years and above. Non-randomised studies (including cohort studies, case–control studies and quasi-randomised studies) assessing the use of cranberry in treating acute UTIs were also eligible. For inclusion, cranberry extract needed to be orally administered as juice, fruit or as capsules/tablets/pills. In studies in which a cranberry product was combined with another intervention/exposure, data allowing the effect of cranberry on the outcome(s) of interest to be isolated were required. Included studies needed to report at least one of our primary or secondary outcomes. Primary outcomes were assessment of participants’ symptoms/clinical status/wellbeing assessment (e.g., symptom burden or time to resolution of symptoms), antibiotic use (immediate and/or delayed) and clinical cure. Secondary outcomes were microbiological cure/assessment; biochemical assessment; assessment of mechanisms of action; and assessment of harms/adverse events.

We excluded studies of exclusively complicated UTI (e.g., catheterised, self-catheterising, spinal cord injury, renal tract abnormalities, male UTIs and pyelonephritis); studies assessing recurrent UTI; animal studies; case reports; and systematic reviews. Systematic reviews were used as sources for references.

### 2.3. Risk of Bias

We assessed the risk of bias of included studies using the Cochrane risk of bias tool [17]. Two reviewers (OAG and EAS) independently assessed the risk of bias of included studies, with disagreements resolved through discussion. The hierarchy of evidence of included studies was classified according to The Oxford Levels of Evidence 2 criteria [18]. 

### 2.4. Data Extraction

We extracted data from included studies on study setting, participants, study duration, the intervention and comparator and the results. We reported the risk of bias across the studies graphically using RevMan [19] and used a summary table to present the results of included studies. The data were independently extracted by two reviewers (OAG and EAS), with disagreements resolved through discussion. We had insufficient data to perform data synthesis and therefore present the results in narrative.

## 3. Results

### 3.1. Study Screening

**Figure 1:**
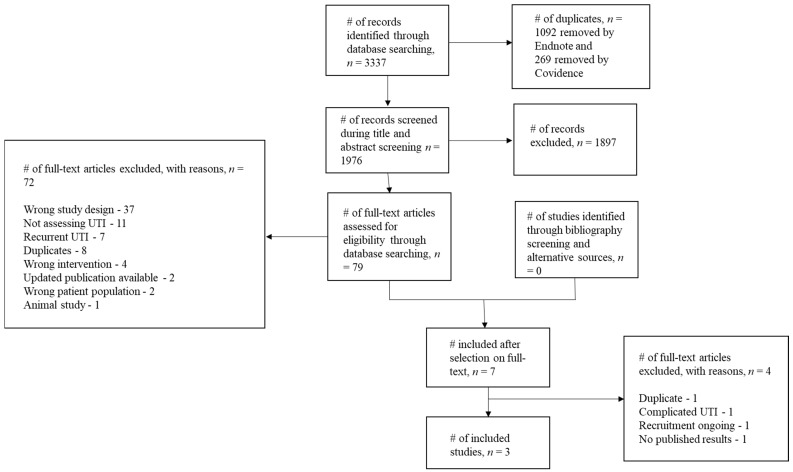
Flow chart showing the process for identification of studies eligible for inclusion.

Electronic database searches identified 3337 results (Figure 1). Searching ClinicalTrials.gov, Google Scholar and WHO ICTRP did not identify additional studies suitable for inclusion. After removal of duplicates, 1976 citations were screened at the title and abstract stage and 79 eligible articles were identified. Thirty seven studies were excluded because the study design did not fit our criteria [12,20,21,22,23,24,25,26,27,28,29,30,31,32,33,34,35,36,37,38,39,40,41,42,43,44,45,46,47,48,49,50,51,52,53,54,55]; a number of these studies assessed the anti-adhesion effects of urine obtained from healthy volunteers following cranberry consumption, when combined with uropathogen cultures ex vivo. Eleven studies were excluded as they did not assess UTI [56,57,58,59,60,61,62,63,64,65,66]. Seven studies were excluded as they assessed recurrent, rather than acute, UTI [67,68,69,70,71,72,73]. Four studies assessed an intervention that did not fit our criteria, such as cranberry extract combined with additional compounds [74,75,76,77]. Two studies were excluded as updated publications were available for eligibility screening [21,78] and a further two studies were excluded as they assessed the wrong patient population [79,80]. One animal study was also excluded [81]. Eight studies were excluded as they were duplicates of five eligible studies [21,36,43,65,68]. After initially including seven studies, a further four were excluded; one was a duplicate [82], the author of a second study confirmed that they included patients with complicated UTI [83], one was a trial registration of an ongoing RCT [82] and one was a completed feasibility RCT with no published results [84]. Three studies were therefore included in this review [85,86,87].

### 3.2. Study Characteristics

All three RCTs were conducted in outpatient settings and included between 60 [87] and 319 [85] participants (Table 1). One study each was conducted in the USA [85], India [87] and the UK [86]. The intervention used in two of the RCTs was cranberry juice [85,86], whilst the other trial used encapsulated cranberry powder [87]. The PAC content of the interventions varied greatly; participants in one study received between 7.5 (low dose) and 15 mg (high dose) of PAC daily [87], whilst participants in another study received on average 224 mg of PAC daily [85]. One study did not report the PAC content in the intervention [86]. 

The included RCTs provided information on outcomes relevant to this review; however, the primary objective of the studies was not assessment of cranberry extract for acute UTI. Two studies focused on cranberry for preventing recurrent UTI [85,87]. Barbosa-Cesnik et al. recruited women with an acute UTI, treating the index UTI with immediate antibiotics and concurrently randomly assigning the participants to receive either 8 ounces of 27% low-calorie cranberry juice twice daily or 8 ounces of placebo juice twice daily for 6 months [85]. Women were followed up for 6 months or until they experienced a UTI, whichever came sooner. Sengupta and colleagues randomly assigned 60 women to receive either low-dose encapsulated cranberry powder (500 mg daily), high-dose encapsulated cranberry powder (1000 mg daily) or no treatment [87]. The primary outcome was the ability of the different treatment regimens to prevent recurrent UTI over a 90-day period.

The primary objective of the trial by Little et al. was to determine the effectiveness of five treatment strategies in the management of suspected acute, uncomplicated UTI, with participants randomly assigned to: (1) immediate antibiotics; (2) delayed antibiotics; (3) antibiotics dependent on the participant’s symptom score; (4) antibiotics offered if the dipstick was positive; and (5) antibiotics targeted to according to midstream urine results [88]. Four forms of self-help advice were randomised across the five groups in a factorial design and included: (1) information leaflet with tips on self-help; (2) advice to use over-the-counter herbal remedies; (3) advice to use bicarbonate; and (4) advice to drink at least 3–4 litres per day and to make at least one litre of this cranberry juice or orange juice.

### 3.3. Risk of Bias

**Figure 2:**
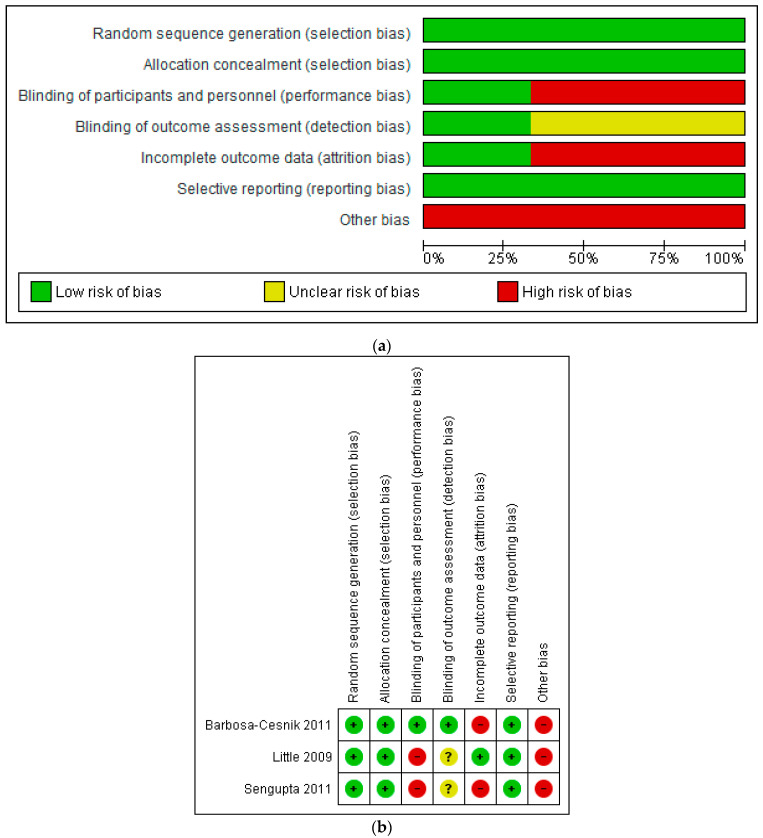
(**a**) Risk of bias graph: review authors’ judgements about each risk of bias item presented as percentages across all included studies. (**b**) Risk of bias summary: review authors’ judgements about each risk of bias item for each included study.

The risk of bias of included studies was judged as moderate (Figure 2a,b), providing level 2 (randomised clinical trial) evidence according to The Oxford Levels of Evidence 2 criteria [18]. All three studies were judged to have a low risk of selection bias and reporting bias. The RCT by Sengupta et al. is described by the study authors as double-blind; however, women in the control group did not receive a placebo [87]. It was therefore judged by the review authors to have a high risk of bias with respect to participant blinding. The open-label RCT by Little et al. was similarly judged to have a high risk of bias for this domain [86]. Two of the three RCTs were judged to have a high risk of bias with respect to incomplete outcome data; Sengupta et al. [87] did not conduct intention to treat (ITT) analyses, whilst Barbosa-Cesnik et al. [85] conducted ITT analysis but had high attrition (26%). 

Additional biases included cranberry industry involvement [85] and insufficient power to detect between-group differences for cranberry comparisons [86,87]. According to the power calculation by Barbosa-Cesnik and colleagues [85], recruiting 120 participants in both arms would have provided the study with 80% power to detect between-group differences, assuming that 30% of participants experienced a UTI during the follow-up period. In order to take into account greater loss to follow-up that might occur in the cranberry group compared with the placebo group, the authors planned to recruit 200 participants per group (400 in total). Although 419 women were randomised, 100 women had negative urine cultures and were therefore not eligible for the study and did not receive cranberry or placebo juice. Therefore, 319 women were included in the ITT analysis—less than the authors had planned. Loss to follow-up was not higher in the cranberry group compared with the placebo group; however, recurrence of UTI occurred in 16.9% of participants—lower than anticipated by the authors—which may have adversely impacted the power of the study.

### 3.4. Symptoms

Little et al. [86] analysed the impact of the different treatment strategies on “frequency symptoms” (day-time and night-time urinary frequency, dysuria and urgency) and “unwell symptoms” (restriction of usual activities, abdominal pain and feeling unwell). There was no significant effect of advice to drink cranberry juice on the severity of frequency symptoms (mean difference (MD) −0.01 (95% CI: −0.37 to 0.34), *p* = 0.94)) or the severity of unwell symptoms (MD 0.02 (95% CI: −0.36 to 0.39), *p* = 0.93)), compared with advice to drink water. Advice to drink cranberry juice compared with water did not affect the duration of symptoms rated moderately bad or worse—that is, rated three or more on a scale of zero to six (incident rate ratio (IRR) 1.18 (95% CI: 1.95 to 1.47), *p* = 0.13)).

Sengupta and colleagues reported a significant within-group improvement in symptoms at 10 days compared with baseline in both the high- and low-dose cranberry intervention groups, but not in the untreated controls [87]. No empirical data were presented to support this finding, nor were between group comparisons reported.

Barbosa-Cesnik et al. [85] reported that at 3 days and at 1–2 weeks after enrolment in the trial, the presence of urinary symptoms and vaginal symptoms was similar between the cranberry and placebo juice groups. No empirical data were presented to support this finding. In this study, all women received immediate antibiotics to treat their index UTI; thus, the findings described represent the effect of cranberry juice in addition to immediate antibiotics.

### 3.5. Antibiotic Use

Little et al. [86] found that advice to consume cranberry juice had no significant impact on the use of antibiotics, compared with advice to drink water (odds ratio (OR) 1.27 (95% CI: 0.47 to 3.43), *p* = 0.64). 

### 3.6. Microbiological Assessment

Sengupta et al. [87] reported a significant within-group reduction in *E. coli* load after 10 days of treatment with both low-dose cranberry (*p* < 0.01) and high-dose cranberry (*p* < 0.0001), but not in the untreated controls (*p* = 0.72). At baseline, 4/13 (30.8%) of the untreated controls were *E. coli* positive, whilst 14/21 (66.7%) of the low-dose cranberry group and 17/23 (73.9%) of the high-dose cranberry group were *E. coli* positive.

### 3.7. Time to Reconsultation

There was no significant impact of cranberry juice consumption on time to re-consultation compared with advice to drink water (hazard ratio (HR) 0.74 (95% CI: 0.49 to 1.13), *p* = 0.17)) in the RCT by Little and colleagues [86].

### 3.8. Serious Adverse Events 

There were no major adverse events (defined as major illness, admission to hospital, death) reported for any group in the trial by Little et al. [86]. Sengupta et al. [87] similarly reported that no serious adverse events occurred during the course of the study. Barbosa-Cesnik and colleagues found that serious adverse events occurred equally between groups, and none were deemed to be related to treatment received in the trial [85]. 

## 4. Discussion

The current evidence base for or against the use of cranberry extract in the management of acute, uncomplicated UTIs is inadequate. The existing, limited RCT evidence identified suggests that advice to consume cranberry juice does not improve urinary frequency symptoms, feeling unwell or the duration of symptoms rated moderately bad or worse in women with acute UTIs, compared with encouraging the consumption of water. Advice to consume cranberry juice did not reduce the use of antibiotics compared with promoting the consumption of water or time to re-consultation. In women receiving immediate antibiotics and cranberry juice, urinary symptoms were not reduced compared with immediate antibiotics and placebo juice. Consuming encapsulated cranberry powder may reduce *E. coli* load and improve symptoms after 10 days of consumption compared with baseline. The studies did not report evidence of serious harm associated with cranberry consumption. These results must be interpreted with caution as they come from a limited number of studies with a moderate risk of bias for the outcomes of interest in this review, which were not the primary objectives of the trials. 

### 4.1. Comparison with Existing Literature

We identified two trial registrations pertinent to this review. One of these trials, an open-label RCT in Spain, aims to recruit 128 women from emergency departments and outpatient clinics with acute UTI to assess the non-inferiority of acute treatment with a Cysticlean cranberry capsule (containing 240 mg PAC) compared with a 3-gram stat dose of Fosfomycin [82]. The primary outcome measures include a comparison of women experiencing “treatment failure” and patient-reported symptoms. The second study is an open-label feasibility RCT in the UK, in which 46 women recruited from GP practices with symptoms suggestive of an acute UTI were randomly assigned to receive: (1) immediate antibiotics; (2) immediate antibiotics and cranberry capsules for up to seven days (72 mg PAC per day); or (3) immediate cranberry capsules for up to seven days (72 mg PAC per day), with a prescription of back-up antibiotics in case symptoms did not improve with cranberry alone, or worsened [84]. The primary outcomes of this feasibility trial relate principally to the ability to recruit participants, the ability to capture data through participant completed symptom diaries and the acceptability of the study procedures and intervention to participants. Dissemination of the findings of these two studies should provide a useful addition to the current, limited evidence base and may serve as a platform for further research assessing cranberry extract as a treatment for symptoms of acute UTI.

A Cochrane review assessing cranberry products for symptoms of acute UTIs, last updated in 2020, did not find any eligible studies, nor did it identify either of the trial registrations discussed above [36]. This review therefore provides additional pertinent information.

Howell et al. [65] conducted a randomised double-blind study to determine the optimal amount of PAC to consume to provide *E. coli* anti-adhesion activity in urine. Urine samples were collected from study participants before and after consuming cranberry capsules containing varying amounts of PAC or placebo capsules. The anti-adhesion activity of the participants’ urine was tested ex vivo against a uropathogenic *E. coli* strain. There was a significant increase in the anti-adhesion activity of PAC compared with placebo (*p* < 0.001), and the effect increased in a dose-dependent fashion. They determined that the optimal amount of PAC to consume was 72 mg per day. In the study by Little et al., the amount of PAC consumed by participants in the cranberry juice arm was not specified, and in the trial by Sengupta et al., participants in the high-dose cranberry group received 15 mg of PAC daily. It is therefore possible that participants in the cranberry arms of these studies were consuming sub-therapeutic doses of what is believed to be the active ingredient in the intervention.

Some studies have found that advice to take non-steroidal anti-inflammatories (NSAIDS) reduces the consumption of antibiotics for acute urinary tract infections, although they control UTI symptoms less effectively than antibiotics and patients had more cases of pyelonephritis [3,4,6,89]. Should cranberry prove to be an effective treatment for acute urinary tract infection, cranberry may confer certain advantages over NSAIDS, such as the appeal of consuming a natural product, as well as additional purported health benefits of cranberry within the urinary tract and elsewhere [80]. Potential harm associated with cranberry consumption, however, must be considered. There is mixed evidence of an interaction between cranberry and Warfarin [90] and of an association with urolithiasis [91]. 

### 4.2. Strengths and Limitations

We employed a broad search strategy to maximise the chance of capturing relevant studies, including grey literature. In addition to electronic database searches and trial registries, we contacted companies that sell cranberry products and experts in the field. When needed, we contacted authors of eligible studies to check whether they were suitable for inclusion. 

However, we recognise that there are limitations to this review. We identified few studies suitable for inclusion, with moderate risk of bias, and empirical data were not provided for all of the outcomes assessed in this review. This is probably in part because cranberry extract as an acute UTI treatment was not the primary focus of the included RCTs. It is possible that we may have missed some studies that were suitable for inclusion, particularly unpublished studies. There was heterogeneity in the outcomes reported by the studies, and in the amount of PAC in the interventions used, making it difficult to make direct comparisons between studies. 

Two of the studies were under-powered to determine the effects of cranberry on outcomes, which can lead to exaggerated effect sizes [92]. One study had high attrition, and another did not conduct intention to treat analyses. 

One study reported within-group comparisons rather than between-group comparison; this can lead to high Type I error (rejection of a true null hypothesis) and misleading results [93]. Whilst Little et al. [86] did not find that cranberry improved UTI symptoms or antibiotic usage, the study authors noted that nearly half of the participants who were advised to drink water alone reported drinking cranberry juice (49%). It is possible that “contamination” of the comparator group may have introduced Type II error (non-rejection of a false null hypothesis), making cranberry juice appear less effective than it is. 

### 4.3. Implications for Future Research and Clinical Practice

Few studies have assessed the utility of cranberry in treating symptoms of acute UTIs; further adequately powered, well-conducted randomised clinical trials are required. These studies should use standardised interventions with a specified amount of PAC and must also report potential harm associated with cranberry consumption. It would also be helpful if the outcomes reported were standardised, to allow direct comparisons to be made between studies and meaningful meta-analysis of multiple studies to be performed. Given that cranberry extract is commonly used by women for symptoms of acute UTI, disseminating the results of well-conducted studies evaluating cranberry extract as a treatment for acute UTI to both clinicians and the public will be important. 

There is a drive towards increasing the use of delayed antibiotic prescription for self-limiting bacterial infections in primary care [94]. In primary care, this strategy has been shown to reduce antibiotic prescription for acute respiratory infections by 40% [95] and for UTI by 20% [96]. If cranberry were found to be effective in robust clinical trials in managing acute uncomplicated UTIs, it could be incorporated into a delayed antibiotic prescribing strategy; women could be advised to take cranberry products initially, taking antibiotics only if symptoms fail to improve or worsen. However, in light of the very limited evidence, no clinical recommendations can be made at present. 

## 5. Conclusions

There is a paucity of studies evaluating cranberry in the management of acute UTIs; none of the identified trials were primarily focused on cranberry as an acute UTI treatment. The existing studies are at moderate risk of bias. Evidence of the effectiveness and safety of cranberry extract as a treatment for symptoms of acute, uncomplicated UTI is inconclusive; rigorous trials addressing these outcomes are required.

## Figures and Tables

**Table 1:** Characteristics of included studies and key results.

Study ID and Country	Design	Participants and Setting	Number of Participants	Age (years)	Study Duration	Intervention	Control	Results
Barbosa-Cesnik et al. (2011), USA [85]	Randomised placebo-controlled trial	Women with an acute UTI (three or more urinary symptoms) presenting for urinalysis at the University of Michigan Health Service laboratory with symptoms of UTI	319 (155 received cranberry, 164 received placebo)	18–40	6 months	8 ounces of 27% low-calorie cranberry juice twice daily	8 ounces of placebo juice twice daily	The presence of urinary and vaginal symptoms was similar between groups at 3 days and at 1–2 weeks.
Little et al. (2009), UK [86]	Randomised controlled trial	Non-pregnant women presenting to General Practices in South-West England with a suspected uncomplicated UTI	309 (241 women in the juice comparisons: 75 advised to take cranberry juice, 78 advised to take orange juice, 88 advised to drink water)	17–70	Average follow-up time of 575 days (range 35–968 days)	Advice to drink cranberry juice	Advice to drink water	No significant impact of advice to consume cranberry juice on the duration of symptoms rated moderately bad or worse (IRR 1.18 (95% CI: 1.95 to 1.47), *p* = 0.13), frequency symptom severity (mean difference −0.01 (95% CI: −0.37 to 0.34), *p* = 0.94), severity of unwell symptoms (mean difference 0.02 (95% CI: −0.36 to 0.39), *p* = 0.93), use of antibiotics (odds ratio 1.27 (95% CI: 0.47 to 3.43) *p* = 0.64) or time to re-consultation (hazard ratio 0.74 (95% CI: 0.49 to 1.13), *p* = 0.17).
Sengupta et al. (2011), India [87]	Randomised controlled trial	Women with mild symptoms of a UTI, urine culture positive and with a negative pregnancy test	60 (16 untreated controls, 21 received low dose cranberry, 23 received high dose cranberry)	18–40	90 days	Encapsulated PAC Standardised Whole Cranberry Powder (PS-WCP)—500 (low dose) and 1000 mg (high dose)	No treatment	Significant within-group improvement of symptoms at day 10 compared to the baseline in both treatment groups, but not in the untreated controls. Significant within-group reduction in *E. coli* load in both treatment groups after 10 days of treatment (low dose, *p* < 0.01; high dose *p* < 0.0001; at a statistical significance level of 95%), but not in the untreated controls (*p* = 0.72).

**Abbreviations:** IRR: incidence rate ratio; PAC: proanthocyanidins; UTI: urinary tract infection.

## Supplementary Materials

The following are available online at https://www.mdpi.com/2079-6382/10/1/12/s1, Table S1: Search strategy.
