# Supplementary material for: Cranberry Extract for Symptoms of Acute, Uncomplicated Urinary Tract Infection: A Systematic Review
Source: Antibiotics (Basel). 2020 Dec 25;10(1):12. doi: 10.3390/antibiotics10010012 (PMC7824375; doi:10.3390/antibiotics10010012)
Supplement: Supplementary file 1 [file antibiotics-10-00012-s001.pdf]

antibiotics

MDPI

Table S1: Search Strategies

Medline

1	urinary tract infections/ or bacteriuria/ or pyuria/	43629
2	("urinary tract infection*" or "urin*" or "urine*" or "UTI*").ti,ab.	1241951
3	Cystitis/	7454
4	Pyelonephritis/	14017
5	cystitis*.ti,ab.	11008
6	pyelonephrit*.ti,ab.	12823
7	Escherichia coli/	263459
8	(bacter* or pyuria or Escherichia*).ti,ab.	996305
9	"E. coli".ti,ab.	131054
10	1 or 2 or 3 or 4 or 5 or 6 or 7 or 8 or 9	2274418
11	Vaccinium macrocarpon/	817
12	(cranberr* or vaccinium).ti,ab.	2688
13	("fruit adj5 juice" or "fruit adj5 drink" or "fruit adj5 beverage").ti,ab.	0
14	exp animals/ not humans.sh.	4669577
15	11 or 12 or 13	2744
16	(10 and 15) not 14	820

Embase

1	urinary tract infections/ or bacteriuria/ or pyuria/	47998
2	("urinary tract infection*" or "urin*" or "urine*" or "UTI*").ti,ab.	1653181
3	Cystitis/	14302
4	Pyelonephritis/	15741
5	cystitis*.ti,ab.	15909
6	pyelonephrit*.ti,ab.	14649
7	Escherichia coli/	335259
8	(bacter* or pyuria or Escherichia*).ti,ab.	1107642
9	"E. coli".ti,ab.	150100
10	1 or 2 or 3 or 4 or 5 or 6 or 7 or 8 or 9	2795171
11	Vaccinium macrocarpon/	1504
12	(cranberr* or vaccinium).ti,ab.	3271
13	("fruit adj5 juice" or "fruit adj5 drink" or "fruit adj5 beverage").ti,ab.	0
14	(exp animals/ or nonhuman/) not human/	6432532
15	11 or 12 or 13	3604
16	conference*.pt.	4461028
17	(10 and 15) not (14 or 16)	834

Amed

1	urinary tract infections/ or bacteriuria/ or pyuria/	168
2	("urinary tract infection*" or "urin*" or "urine*" or "UTI*").ti,ab.	9588
3	Cystitis/	58
4	Pyelonephritis/	8
5	cystitis*.ti,ab.	80
6	pyelonephrit*.ti,ab.	18
7	(bacter* or pyuria or Escherichia*).ti,ab.	2020
8	"E. coli".ti,ab.	144
9	1 or 2 or 3 or 4 or 5 or 6 or 7 or 8	11637
10	(cranberr* or vaccinium).ti,ab.	113
11	("dietary supplement*" or "herbal adj5 supplement" or "food adj5 supplement" or "fruit adj5 juice" or "fruit adj5 drink" or "fruit adj5 beverage").ti,ab.	541
12	exp animals/ not humans.sh.	12241
13	10 or 11	650
14	(9 and 13) not 12	87

Cinahl

	(MH "Urinary Tract Infections") OR (MH "Bacteriuria") OR (MH "Pyuria") OR (("urinary tract infection*" or "urin*" or "urine*" or "UTI*") OR cystitis OR pyelonephritis OR "Escherichia coli" OR "E.coli"	
AND	("fruit N5 juice" or "fruit N5 drink" or "fruit N5 beverage") OR "Vaccinium macrocarpon" OR cranberr* OR vaccinium	
NOT	(MH "Animals+") NOT (MH Humans)	517

Cochrane

1	(Title/abstract/keyword search)	urinary tract infection OR UTI OR bacteria OR bacteriuria OR pyuria OR urine OR cystitis OR pyelonephritis OR "Escherichia coli" OR "E.coli"
2	(Title/abstract/keyword search)	cranberry OR vaccinium

	1 AND 2	247 trials, 6 reviews

Web of Science

# 5	826	#3 NOT #4
		Indexes=SCI-EXPANDED, SSCI, A&HCI, CPCI-S, CPCI-SSH, BKCI-S, BKCI-SSH, ESCI, CCR-EXPANDED, IC Timespan=All years
# 4	7,366,900	TS=(animals or animal or mice or mus or mouse or murine or woodmouse or rats or rat or murinae or muridae or cottonrat or cottonrats or hamster or hamsters or cricetinae or rodentia or rodent or rodents or pigs or pig or swine or swines or piglets or piglet or boar or boars or sus scrofa or ferrets or ferret or polecat or polecats or mustela putorius or guinea pigs or guinea pig or cavia or callithrix or marmoset or marmosets or cebuella or hapale or octodon or chinchilla or chinchillas or gerbillinae or gerbil or gerbils or jird or jirds or merione or meriones or rabbits or rabbit or hares or hare or diptera or flies or fly or dipteral or drosphila or drosophilidae or cats or cat or carus or felis or nematoda or nematode or nematoda or nematode or nematodes or sipunculida or dogs or dog or canine or canines or canis or sheep or sheeps or mouflon or mouflons or ovis or goats or goat or capra or capras or rupicapra or chamois or haplorhini or monkey or monkeys or anthropoidea or anthropoids or saguinus or tamarin or tamarins or leontopithecus or hominidae or ape or apes or pan or paniscus or pan paniscus or bonobo or bonobos or troglodytes or pan troglodytes or gibbon or gibbons or siamang or siamangs or nomascus or symphalangus or chimpanzee or chimpanzees or prosimians or bush baby or prosimian or bush babies or galagos or galago or pongidae or gorilla or gorillas or pongo or pygmaeus or pongo pygmaeus or orangutans or pygmaeus or lemur or lemurs or lemuridae or horse or horses or pongo or equus or cow or calf or bull or chicken or chickens or gallus or quail or bird or birds or quails or poultry or poultries or fowl or fowls or reptile or reptilia or reptiles or snakes or snake or lizard or lizards or alligator or alligators or crocodile or crocodiles or turtle or turtles or amphibian or amphibians or amphibia or frog or frogs or bombina or salientia or toad or toads or epidalea calamita or salamander or salamanders or eel or eels or fish or fishes or pisces or catfish or catfishes or siluriformes or arius or heteropneustes or sheatfish or perch or perches or percidae or perca or trout or trouts or char or chars or salvelinus or fathead minnow or minnow or cyprinidae or carps or carp or zebrafish or zebrafishes or goldfish or goldfishes or guppy or guppies or chub or chubs or tinca or barbels or barbus or pimephales or promelas or poecilia reticulata or mullet or mullets or seahorse or seahorses or mugil curema or atlantic cod or shark or sharks or catshark or anguilla or salmonid or salmonids or whitefish or whitefishes or salmon or salmons or sole or solea or sea lamprey or lamprey or lampreys or pumpkinseed or sunfish or sunfishes or tilapia or tilapias or turbot or turbots or flatfish or flatfishes or sciuridae or squirrel or squirrels or chipmunk or chipmunks or suslik or susliks or vole or voles or lemming or lemmings or muskrat or muskrats or lemmus or otter or otters or marten or martens or martes or weasel or badger or badgers or ermine or mink or minks or sable or sables or gulo or gulos or wolverine or wolverines or minks or mustela or llama or llamas or alpaca or alpacas or camelid or camelids or guanaco or guanacos or chiroptera or chiropteras or bat or bats or fox or foxes or iguana or iguanas or xenopus laevis or parakeet or parakeets or parrot or parrots or donkey or donkeys or mule or mules or zebra or zebras or shrew or shrews or bison or bisons or buffalo or buffaloes or deer or deers or bear or bears or panda

		or pandas or wild hog or wild boar or fitchew or fitch or beaver or beavers or jerboa or jerboas or capybara or capybaras)
		Indexes=SCI-EXPANDED, SSCI, A&HCI, CPCI-S, CPCI-SSH, BKCI-S, BKCI-SSH, ESCI, CCR-EXPANDED, IC Timespan=All years
# 3	988	#2 AND #1
		Indexes=SCI-EXPANDED, SSCI, A&HCI, CPCI-S, CPCI-SSH, BKCI-S, BKCI-SSH, ESCI, CCR-EXPANDED, IC Timespan=All years
# 2	9,076	TS=("fruit near/5 juice" OR "fruit near/5 drink" OR "fruit near/5 beverage" OR "vaccinium macrocarpon" OR cranberr* OR vaccinium)
		Indexes=SCI-EXPANDED, SSCI, A&HCI, CPCI-S, CPCI-SSH, BKCI-S, BKCI-SSH, ESCI, CCR-EXPANDED, IC Timespan=All years
# 1	954,458	TS=("Urinary Tract Infection" OR urin* OR urine* OR UTI OR cystitis OR pyelonephritis OR "Escherichia coli" OR "E. coli")
		Indexes=SCI-EXPANDED, SSCI, A&HCI, CPCI-S, CPCI-SSH, BKCI-S, BKCI-SSH, ESCI, CCR-EXPANDED, IC Timespan=All years
